# Delivery Induced Intraperitoneal Rupture of a Cystic Ovarian Teratoma and Associated Chronic Chemical Peritonitis

**DOI:** 10.1155/2014/189409

**Published:** 2014-03-12

**Authors:** Reine Nader, Thibault Thubert, Xavier Deffieux, Jocelyne de Laveaucoupet, Guillaume Ssi-Yan-Kai

**Affiliations:** ^1^AP-HP, Service de Radiologie, Hôpital Antoine Béclère, 157 rue de la Porte de Trivaux, 92141 Clamart, France; ^2^AP-HP, Service de Gynécologie-Obstétrique et Médecine de la Reproduction, Hôpital Antoine Béclère, 92141 Clamart, France

## Abstract

Intraperitoneal rupture of cystic ovarian teratoma is a rare complication. We report a case in a 29-year-old female, with increased abdominal circumference 2 months after vaginal delivery. MRI/CT raised this diagnosis associated to chemical peritonitis. A malignant ovarian mass with peritoneal carcinomatosis was excluded. Laparoscopic oophorectomy was performed and histologic analysis confirmed imaging findings. This case demonstrates the interest of imaging before surgery in pelvic masses to avoid misdiagnosing and to provide adequate treatment.

## 1. Introduction

Mature cystic teratoma of the ovary is the most common ovarian neoplasm, accounting for between 5 and 25% of all ovarian tumors. It occurs most commonly in young females and is bilateral in 8–15% of cases. It comprises a cyst lined by an epidermis-like epithelium and contains a variable admixture of elements of one or more of the three cell lines, meso-, endo-, and ectodermal derivatives including sebaceous secretions, hair, teeth, bone, or fat, and is asymptomatic in most of cases; however, it may represent serious complications including torsion (16%), followed by spontaneous rupture (1.3%) and infection (1.2%) and rarely malignant degeneration and hemolytic anemia.

## 2. Case Report

A 29-year-old female patient, gravid 2, para 2, was addressed to our radiological department by her gynecologist for investigation of a left ovarian mass and increased abdominal circumference 2 months after normal vaginal delivery.

**Figure 1:**
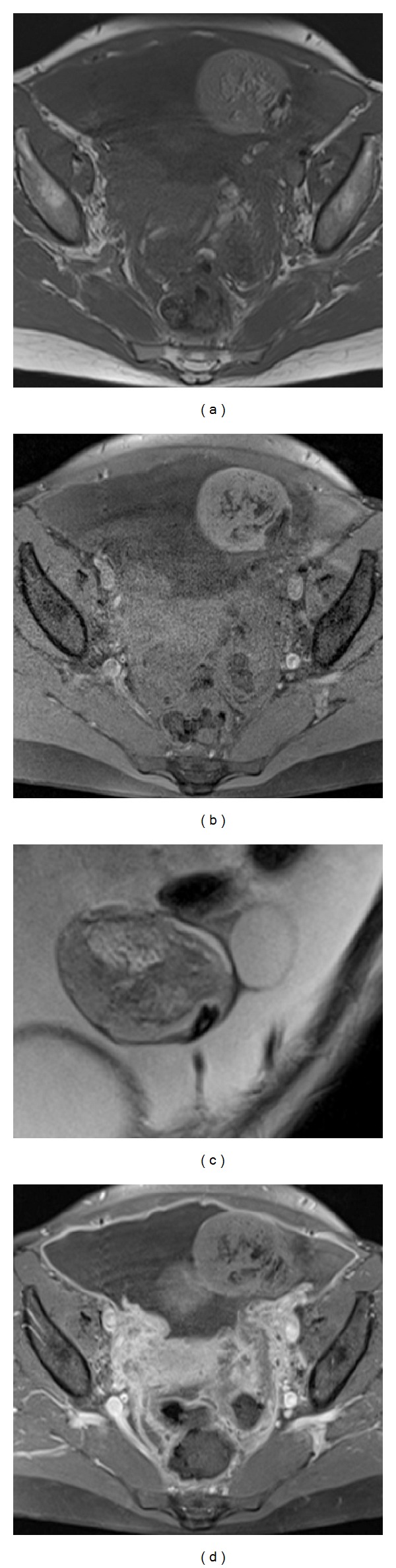
Axial T1 weighted (a) and with fat saturation (b). Images show a large heterogenous left ovarian mass measuring with fatty, solid, and liquid contents and a small calcification on coronal T2 weighted image (c) suggestive of cystic teratoma. Axial T1 postcontrast image (d) demonstrates ascites with peritoneal thickening.

MRI was obtained and showed a large heterogenous left ovarian mass measuring 85 × 50 × 45 mm with fatty, solid, and liquid contents and a small calcification of 10 mm suggestive of cystic teratoma (Figure 1). Ascites and peritoneal thickening were also detected with fat globules in the cul de sac.

**Figure 2:**
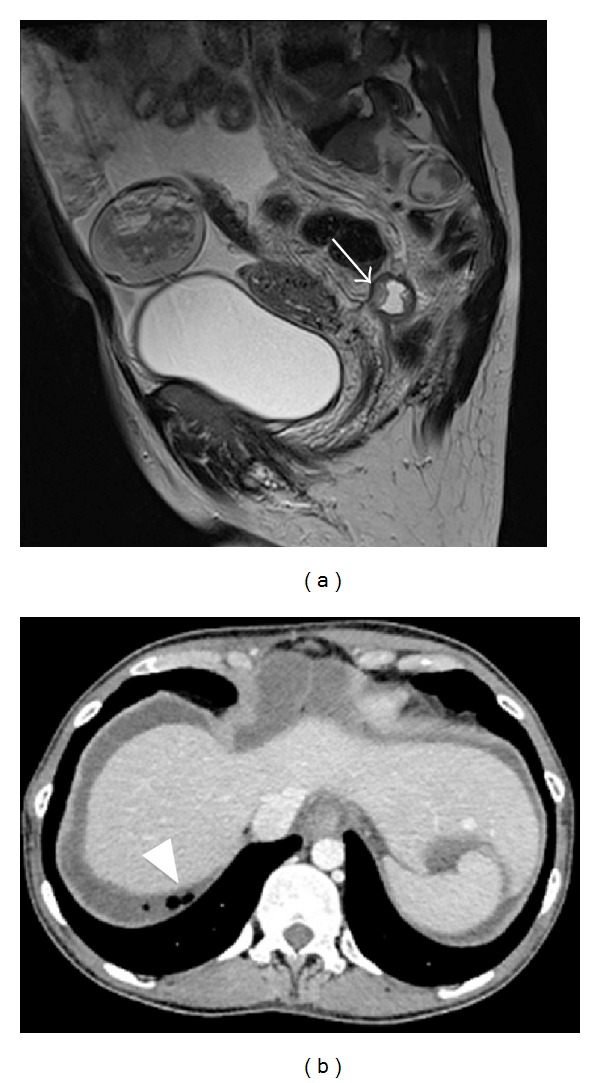
Sagittal T2 weighted image (a) and axial CT (b) demonstrate intraperitoneal rupture with fat globules in the cul de sac (arrow) and below the right hemidiaphragm (arrowhead), a pathognomonic finding.

A CT scan was also obtained to confirm the diagnosis of delivery induced intraperitoneal rupture of a cystic ovarian teratoma and associated chronic chemical peritonitis (Figure 2).

**Figure 3:**
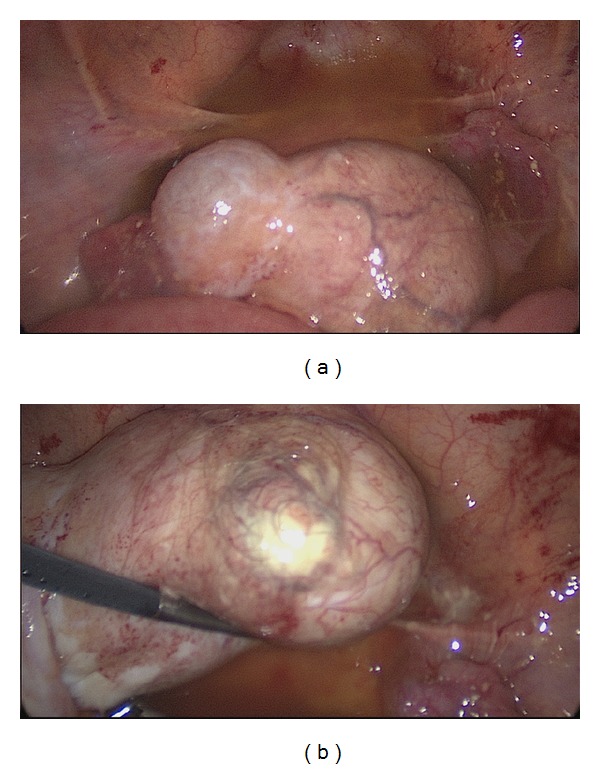
Laparoscopic view shows dense adhesions and the thick white to yellowish plaque-like lesion on the visceral peritoneum (a) and a huge ovarian mass consistent with a ruptured mature cystic teratoma.

The patient underwent laparoscopic oophorectomy. Lyses of the dense adhesions and the thick white to yellowish plaque-like lesion on the visceral peritoneum, especially on the surface of the uterus and rectum, were performed with copious saline washing of the chemical peritonitis and its sequelae (Figure 3).

The patient had an uneventful recovery.

## 3. Discussion

The word teratoma is derived from the Greek word Terato meaning monster.

Mature cystic teratomas are composed of well-differentiated derivations from at least two of the three germ cell layers (ectoderm, mesoderm, and endoderm). They therefore contain developmentally mature skin complete with hair follicles and sweat glands, sometimes luxuriant clumps of long hair, and often pockets of sebum, blood, fat, bone, nails, teeth, eyes, cartilage, and thyroid tissue. They are usually asymptomatic but can complicate and sometimes lead to fatal consequences if not adequately treated [1, 2].

Some of the complications are as follows [1–4].

- The adnexal torsion considered as the most common complication caused by rotation of the ovarian pedicle, resulting in arterial, venous, or lymphatic obstruction and involves the ovary and the fallopian tube rather than either alone. US is the first examination for diagnosing adnexal torsion in an emergency setting. If diagnosis and reduction of ovarian torsion are delayed, hemorrhagic infarction occurs and sometimes leads to severe peritonitis and even death.
- Malignant transformation occurs in 1%-2% of ovarian teratomas and accounts for 1% of all ovarian malignancies and occurs usually in patients of more than 45 years. It may occur in any of the three germ cell layers including the ectoderm, mesoderm, and endoderm. Squamous cell carcinoma arising from the squamous lining of the cyst is the most common type of malignant transformation, accounting for 80% of the reported cases.
- Rupture occurs in 1%–4% of ovarian teratomas and causes leakage of the liquefied sebaceous contents into the peritoneum, which irritates the peritoneum and leads to acute or chronic inflammation.
- Superimposed infection occurs in 1% with Coliform bacteria most commonly implicated.
- Autoimmune haemolytic anaemia occurs in <1% and may be due to cross-reactivity of tumor and red blood cell antigens, production of red blood cell autoantibodies by the tumor, and alteration of the red blood cell molecules by the tumor, which renders them antigenic to the host.

### 3.1. Chemical Peritonitis [3–5]

Chemical peritonitis is a result of an intraperitoneal rupture of a dermoid cyst. First case described in 1843 by Barth was of a 40-year-old female presenting with fat globules adherent to the liver and associated with an ovarian tumor containing fat and hair. In 1952, Geist was the first to describe the X-ray findings of multiple abdominal calcified lesions and thus diagnosis of a ruptured intraperitoneal ovarian cyst. Spontaneous rupture is an extremely rare complication of mature cystic teratoma because of its usually thick capsule. The cause of rupture may be due to torsion with infarction of the tumor, infection, malignancy and rapid growth of the cyst, direct trauma, or prolonged pressure from pregnancy or delivery as in our case.

It can occur in the peritoneal cavities or, less frequently, into the adjacent hollow viscus, such as the bladder, small bowel, rectum, sigmoid colon, vagina, and even through abdominal wall resulting in the following.

Acute peritonitis due to rupture and sudden release of tumour contents rupture that may result in acute abdominal crisis, shock, or hemorrhage.

Chronic granulomatous peritonitis, which is more common, is due to a chronically leaking dermoid from a tiny breach in the cyst wall, characterized by numerous nodules of mature glial tissue implant on a widespread area of the peritoneum and dense adhesions and variable ascites that simulate carcinomatosis or tuberculous peritonitis. Fluid collection can also occur in the bilateral, paracolic gutters and between the mesenteric leaflets.

The symptoms and signs might be subtle and marginal in the early period; however, the patient would complain of progressive abdominal distention, low abdominal pain, and gastrointestinal disturbances such as anorexia, nausea, vomiting, and diarrhea.

### 3.2. Imaging of Ovarian and Ruptured Dermoids [3]

- * Plain Film.* Plain film may show calcific and tooth components with the pelvis and in the abdomen in case of a ruptured cyst.
- * Ultrasound.* Ultrasound is the most common imaging modality, with 58% sensitivity and 99% specificity in the diagnosis of a mature cystic teratoma, calcified structures, hair, echogenic sebaceous material, and fat contents. It confirms presence of mass, identifies organ of origin and internal structures, and uses Doppler to assess for flow; however, it is limited by abnormal pelvic anatomy and difficult to appreciate cystic quality of these tumors.
- * CT Imaging.* CT imagining has 98% sensitivity and 100% specificity in the diagnosis of a mature cystic teratoma and fat detection (density less than 20 HU). It is diagnostic, gravity dependent layering with fat fluid line, palm tree like protrusion, and fat-fluid levels (10%).
- In case of a ruptured cyst and chemical peritonitis, ascites and floating areas of fat attenuation around the liver as well a mature cystic teratoma of the ovary are seen. Discontinuity of the cyst wall with surrounding infiltration is evident. The characteristic hypoattenuating fatty fluid can be found as antedependent pockets, typically below the right hemidiaphragm, a pathognomonic finding.
- The escaped cyst content also leads to a chemical peritonitis with ascites, diffuse, or focal omental infiltration and inflammatory masses involving the omentum and bowel, which may mimic peritoneal carcinomatosis.
- * MRI Imaging.* MRI imaging has excellent sensitivity for detecting fat and calcification and is useful for detecting organ of origin if ultrasound is nondiagnostic. T1 Weighted: sebum/fat has very high signal intensity; calcium bone and hair are low. T1 Weighted with fat saturation: suppression of high signal sebum/fat is diagnostic. Blood products in hemorrhagic cysts should not suppress. In case of a ruptured cyst, ascites and peritoneal thickening are detected with fat globules in the cul de sac and in the antedependent pockets with a deformed ovarian dermoid cyst.

### 3.3. Treatment

The treatment of choice, once rupture of an ovarian cystic teratoma is diagnosed, is surgical intervention. The cases of spontaneously ruptured ovarian cystic teratoma have a favorable prognosis if obvious intraoperative signs of peritonitis are not seen, because prompt removal of a spontaneously ruptured ovarian cyst with thorough peritoneal lavage is sufficient to prevent prolonged chemical peritonitis.

## 4. Conclusion

Chemical peritonitis is a rare condition that occurs secondary to a spontaneous rupture of a dermoid cyst in the acute presentation, or secondary to a tiny perforation and leakage from a breach in the cyst wall in the chronic form. In the chronic phase, the diagnosis is usually incidental following abdominal discomfort or increase circumference. Intra-abdominal peritoneal seedlings, adhesions, and/or masses are frequent sequelae.

The diagnosis should be raised in front of every case of unexplained chemical peritonitis especially when associated with dermoid cyst or fat globules and/or calcified hepatic or peritoneal deposits. Recognition of a dermoid tumour associated with glial seedling and a good understanding of the imaging findings and of the complications of ovarian teratomas are important to prevent misdiagnosis, to avoid unnecessary debulking surgery, and to achieve adequate treatment.
